# Clinical Features and HLA Genetics Differ in Children at Type 1 Diabetes Onset by Hispanic Ethnicity

**DOI:** 10.1210/clinem/dgae608

**Published:** 2024-09-04

**Authors:** Kagan E Karakus, Theodore Fleury, Erin E Baschal, Kristen A McDaniel, Hyelin Choi, Taylor K Armstrong, Liping Yu, Kimber M Simmons, Aaron W Michels

**Affiliations:** Barbara Davis Center for Diabetes, University of Colorado, Aurora, CO 80045, USA; Barbara Davis Center for Diabetes, University of Colorado, Aurora, CO 80045, USA; Barbara Davis Center for Diabetes, University of Colorado, Aurora, CO 80045, USA; Barbara Davis Center for Diabetes, University of Colorado, Aurora, CO 80045, USA; Barbara Davis Center for Diabetes, University of Colorado, Aurora, CO 80045, USA; Barbara Davis Center for Diabetes, University of Colorado, Aurora, CO 80045, USA; Barbara Davis Center for Diabetes, University of Colorado, Aurora, CO 80045, USA; Barbara Davis Center for Diabetes, University of Colorado, Aurora, CO 80045, USA; Barbara Davis Center for Diabetes, University of Colorado, Aurora, CO 80045, USA

**Keywords:** type 1 diabetes, HLA genetics, islet autoantibodies, ethnicity, Hispanic, diabetic ketoacidosis

## Abstract

**Context:**

Type 1 diabetes incidence continues to increase in children, especially among Hispanic White (HW) children.

**Objective:**

We investigated the clinical, immunologic, and genetic characteristics of HW and non-Hispanic White (NHW) children who presented at type 1 diabetes diagnosis.

**Methods:**

In this single-center, observational study, children who were diagnosed with type 1 diabetes (≤20 years old) and tested for islet autoantibodies within 1 year of diagnosis were included in the study and divided into 2 groups by Hispanic ethnicity.

**Results:**

Of 1297 children, 398 HW children presented with a younger age at diabetes onset (10.2 ± 3.9 vs 11.1 ± 4.1 years, *P* < .001) and more diabetic ketoacidosis (62.4% vs 51.9%, *P* < .001) than NHW children (n = 899). There was no difference in sex, A1c levels, or the number and prevalence of islet autoantibodies between the 2 cohorts. A subset of our cohort was human leukocyte antigen (HLA) typed as specific alleles confer strong genetic risk for type 1 diabetes (eg, HLA-DR4 and DQ8). Among 637 HLA-typed children, HW children had a significantly higher prevalence of the DR4-DQ8 haplotype than NHW children (79.1% vs 60.1%, *P* < .001), and this frequency was much higher than a reference Hispanic population (OR 6.5, 95% CI 4.6-9.3).

**Conclusion:**

Hispanic White children developing type 1 diabetes have a high prevalence of HLA DR4-DQ8, which can be utilized to select individuals for immune monitoring with islet autoantibodies to lessen diabetic ketoacidosis and potentially prevent diabetes onset.

Non-Hispanic White (NHW) children are the predominant race/ethnicity group who develop type 1 diabetes, the immune-mediated form of diabetes, in the United States followed by Hispanic and non-Hispanic Black children (1-3). The SEARCH for Diabetes in the Youth (SEARCH) study, a large multicenter study among an ethnically diverse population of children and adolescents with diabetes, recently showed that Hispanic children have 3- to 4-fold higher annual incidence rates of type 1 diabetes than NHW children (3, 4). Thus, Hispanic children are the second most affected ethnicity developing type 1 diabetes with expanding prevalence in the United States (5). Moreover, higher rates of diabetic ketoacidosis (DKA) have been reported in Hispanic children than in NHW children from multiple studies (6-9). With a rapidly rising incidence and high DKA rates at clinical onset, there is a need to thoroughly evaluate the clinical, immunologic, and genetic features of Hispanic children presenting with newly diagnosed type 1 diabetes. Several studies have been undertaken to measure a subset of islet autoantibodies, such as those directed against glutamic acid decarboxylase and islet antigen-2 and perform partial human leukocyte antigen (HLA) typing in Hispanic youth (7, 10, 11). However, a comprehensive analysis of all 4 biochemical islet autoantibodies along with high-resolution HLA typing for all class II and I alleles has not been evaluated in Hispanic children having type 1 diabetes. Understanding the similarities and differences between NHW and Hispanic White (HW) children with type 1 diabetes in terms of islet autoantibody profiles and HLA genetics holds the potential to improve screening efforts to lessen DKA and potentially prevent diabetes onset (12).

Over the last 3 decades, we identified a large cohort of NHW and HW children presenting with new-onset type 1 diabetes at our diabetes center. Along with demographic data, these children had the 4 biochemical islet autoantibodies measured (eg, those directed against insulin, glutamic acid decarboxylase, islet antigen-2, and zinc transporter 8) and HLA typing for class II and I alleles. We show that HW children have a younger age at type 1 diabetes onset and more DKA than NHW children. Islet autoantibody profiles in terms of prevalence for each autoantibody and overall number did not overly differ by ethnicity. Notably, HW children had a higher prevalence of the diabetes risk HLA-DR4-DQ8 haplotype than NHW children, being present in 80% of HW children with new-onset type 1 diabetes, which is much higher than in the general Hispanic population. These results have important implications for understanding type 1 diabetes pathogenesis across ethnicities and developing screening paradigms to assess diabetes risk which can lower DKA rates.

## Materials and Methods

### Study Design

Subjects were recruited from the Barbara Davis Center for Diabetes clinics, a major referral center for children with new-onset type 1 diabetes, between January 1, 1996, and December 31, 2023. Children with type 1 diabetes who were younger than 20 years of age and had been tested for diabetes autoantibodies within 1 year of clinical diagnosis were included in the study (n = 1297) (Supplementary Dataset (13)). All children were diagnosed with type 1 diabetes using the American Diabetes Association diagnostic criteria (14). Demographic characteristics were collected from a research database and electronic medical records, and participants self-reported race and ethnicity. DKA was defined as ketoacidosis requiring hospitalization and determined from electronic medical records. Islet autoantibody measurements and HLA typing were done on peripheral blood after written informed consent was collected from all participants, or the guardian when the participant was less than 18 years of age. The Colorado Multiple Institutional Review Board approved the study.

### Islet Autoantibody Measurements

Islet autoantibodies were measured from serum using fluid-phase radiobinding assays to insulin (IAAs), glutamic decarboxylase (GADAs), tyrosine phosphatase-related islet antigen-2 (IA-2As), and zinc transporter 8 (ZnT8As) as previously described (15-17). ZnT8A levels were measured from samples beginning in 2010 and later. Harmonized assays for GADA and IA-2A were available starting in 2010, and index values obtained before this time were converted to digestive and kidney units (DK units). The positive cutoff values for each islet autoantibody include an index of 0.010 for IAA, 20 DK units for GADA, 5 DK units for IA-2A, and an index of 0.020 for ZnT8A. IAAs were only considered positive if measured within 21 days of type 1 diabetes diagnosis as exogenous insulin can induce IAAs (18).

### HLA Typing

Stored DNA samples from study participants were used to type HLA alleles (n = 637). HLA typing for HLA-DRB1, DQA1, and DQB1 alleles was performed using oligonucleotide probes for approximately half of the HLA typed individuals, n = 330 (19).The other half (n = 307) of the individuals had HLA class I (A, B, and C) and class II alleles (DQA1, DQB1, DRB1, DPA1, and DPB1) sequenced using a targeted next generation sequencing assay with hybrid capture technology (AlloSeq Tx17 from CareDx Lab Solutions, Inc.) as previously described (20). Initially, 537 samples were randomly chosen for HLA typing (436 NHW, 101 HW). A validation cohort of an additional 100 HW children were randomly selected from the study group and HLA typed.

### Statistical Analysis

Statistical analyses were performed using GraphPad Prism version 9.2 and IBM's SPSS version 29.0 software. Categorical variables (sex, DKA, season at diagnosis, islet autoantibody positivity and number, and HLA type) were compared using Fisher's exact test, and continuous variables (age, A1c, body mass index [BMI], diabetes duration) were compared by ethnicity with independent sample t tests. Islet autoantibody levels were compared using Mann–Whitney U tests between ethnic groups. A logistic regression model assessed the presence of DKA at diagnosis and associated factors. In the model, DKA was the dependent variable and independent variables were ethnicity (NHW/HW), sex, age, A1c, BMI at diagnosis, presence of HLA DR3-DQ2, DR4-DQ8, IAA, GADA, IA2A, ZnT8A, and the number of positive antibodies. To determine season of type 1 diabetes diagnosis, diagnosis dates were categorized into 4 seasons (autumn, winter, spring, and summer) based on the equinox and solstices for each year (21). For HLA typing, we initially typed NHW children (n = 436) and HW children (n = 101). To validate the initial HLA typing results for DR4-DQ8 in HW children, we randomly selected and typed an additional group of HW children (n = 100, validation group) and compared results of the validation group to the initial HW group. We also compared frequencies of HLA genes in our NHW and HW populations with Caucasian and Hispanic reference populations from a national bone marrow transplant registry (22), and reported odds ratios (OR) with 95% CI. *P* < .05 was considered to be statistically significant.

## Results

### Clinical Characteristics of Type 1 Diabetes Participants at Onset by Ethnicity

**Figure 1.**
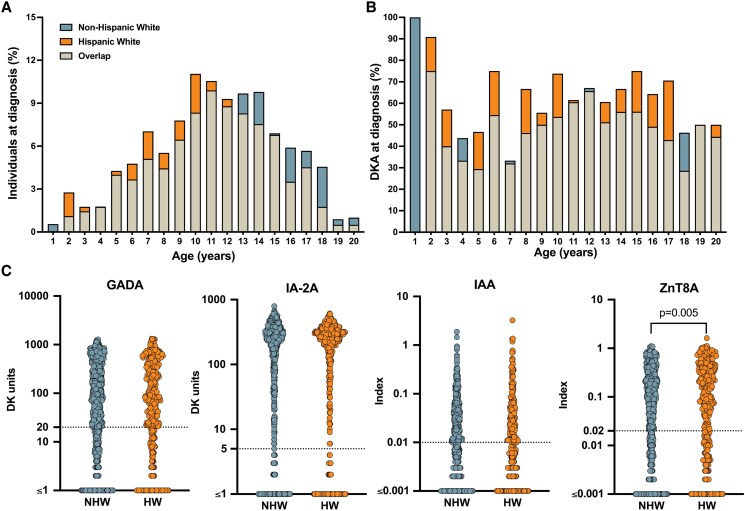
Clinical and immunologic characteristics at type 1 diabetes diagnosis by ethnicity. (A) Distribution of age at diagnosis by ethnicity. (B) Presence of DKA at diagnosis by ethnicity. (C) Islet autoantibody levels for glutamic acid decarboxylase 65 autoantibodies (GADAs), tyrosine phosphatase-related islet antigen-2 autoantibodies (IA-2As), insulin autoantibodies (IAAs), and zinc transporter 8 autoantibodies (ZnT8As). Insulin autoantibody values were included if measured within 21 days of type 1 diabetes diagnosis. Dotted line is at the cutoff for each antibody positivity. *P* = .005 for ZnT8A using a Mann–Whitney U test; *P* = n.s. for the other autoantibodies.

Between March 1996 and December 2023, we identified 1297 children less than 20 years of age with newly diagnosed type 1 diabetes who had a reported race of White and ethnicity that was either non-Hispanic or Hispanic. This resulted in 69% (899/1297) of the participants being NHW and 31% (398/1297) being HW children (Table 1). Most of the group was seen within a week of diabetes diagnosis (78%) with a median time of 2 days. In Table 1, we compare the 2 cohorts by ethnicity, which indicated a younger age at onset in HW children than NHW children (10.2 ± 3.9 years vs 11.1 ± 4.1 years respectively, *P* < .001) and no differences in sex; however, there was a slight male predominance among both ethnicities. The younger age among HW children was driven by a higher frequency of diagnoses among those ages 2 to 12 years and more adolescent NHW cases (Fig. 1A). HW children had a slightly higher BMI at diagnosis (18.5 ± 4.6 vs 17.9 ± 4.1 kg/m^2^, *P* < .001) than NHW children (Table 1).

**Table 1.** Clinical, metabolic, and immunologic characteristics of children with new-onset type 1 diabetes by ethnicity

	All new-onset (n = 1297)	Non-Hispanic White (n = 899)	Hispanic White (n = 398)	*P* value
Age, years, mean ± SD	10.8 ± 4.0	11.1 ± 4.1	10.2 ± 3.9	<.001
Sex, % female (n)	45.2 (586)	43.8 (394)	48.2 (192)	.141
BMI, kg/m^2^, mean ± SD	18.1 ± 4.3	17.9 ± 4.1	18.5 ± 4.6	<.001
DKA, % (n)	55.0 (678)	51.9 (447)	62.4 (231)	<.001
**Season at diagnosis**, % (n)				
Autumn	26.1 (338)	27.3 (245)	23.4 (93)	.141
Winter	27.5 (357)	27.7 (249)	27.1 (108)	.835
Spring	24.4 (316)	23.6 (212)	26.1 (104)	.324
Summer	22.1 (286)	21.5 (193)	23.4 (93)	.447
Type 1 diabetes duration, days, median (IQR)	2 (1-6)	3 (1-6)	2 (0-6)	.869
A1c, %, mean ± SD	12.0 ± 2.4	12.0 ± 2.5	12.2 ± 2.3	.229
A1c, mmol/L, mean ± SD	108 ± 26	108 ± 27	110 ± 25	.229
**Islet autoantibody status**, % (n)				
IAA*^a^*	51.2 (571)	51.0 (390)	51.4 (181)	.949
GADA	70.9 (920)	70.1 (630)	72.9 (290)	.321
IA-2A	74.4 (965)	73.0 (656)	77.6 (309)	.085
ZnT8A	67.4 (785)	66.2 (538)	70.2 (247)	.196
**Number of islet autoantibodies**, %				
0 Ab	6.1 (79)	6.3 (57)	5.5 (22)	.617
1 Ab	13.6 (176)	13.5 (121)	13.8 (55)	.861
2 Ab	25.4 (330)	27.6 (248)	20.6 (82)	.009
3 Ab*^b^*	34.8 (443)	33.3 (295)	38.0 (148)	.110
4 Ab*^c^*	26.7 (269)	25.7 (178)	28.9 (91)	.318

Abbreviations: Ab, autoantibody; BMI, body mass index; DKA, diabetic ketoacidosis; GADA, glutamic decarboxylase autoantibody; IAA, insulin autoantibody; IA-2A, tyrosine phosphatase-related islet antigen-2 autoantibody; IQR, interquartile range; ZnT8A, zinc transporter 8 autoantibody.

^*a*^Insulin antibody positive values were excluded from analysis if measured >21 days after type 1 diabetes diagnosis.

^*b*^n = 1274 measured for at least 3 islet Abs.

^*c*^n = 1007 measured for 4 islet Abs.

As there is a seasonality to type 1 diabetes diagnosis (23), we evaluated the seasons in which NHW and HW children were diagnosed. As expected, there was a higher incidence of all diabetes diagnoses in the winter than in other seasons, but no differences based upon ethnicity (Table 1). Next, we evaluated DKA at diagnosis finding a higher percentage of HW children having DKA than NHW children (62% vs 52% respectively, *P* < .001). Notably, the higher prevalence of DKA in HW children spanned nearly all ages compared with NHW children (Fig. 1B). A1c values were similar between the 2 cohorts (Table 1). We evaluated other associated factors for DKA using a binary logistic regression model, assessing for ethnicity, sex, age, A1c, BMI, presence of IAA, GADA, IA-2A, and ZnT8A, number of positive islet autoantibodies, and the presence of type 1 diabetes risk HLA class II haplotypes DR4-DQ8 and DR3-DQ2 among 533 children who had all of these metrics. DKA was associated with Hispanic ethnicity (OR 1.62, 95% CI 1.05-2.50, *P* = .029), male sex (OR 1.71 95% CI 1.15-2.54, *P* = .008), A1c (OR 1.41 95% CI 1.29-1.54, *P* < .001), and positivity for IAA (OR 1.59, 95% CI 1.01-2.49, *P* = .044).

Taken together, our large cohort of children developing new-onset type 1 diabetes indicates clinical differences between NHW and HW children with regard to a younger age at onset, higher BMI, and more DKA in HW children.

### Islet Autoantibody Profiles

The vast majority of children were positive for at least 1 of the 4 biochemical islet autoantibodies at clinical type 1 diabetes diagnosis (95%), and this did not differ by ethnicity as positivity for IAA, GADA, IA-2A, and ZnT8A was similar between the 2 cohorts (Table 1); IAAs were only considered positive if measured within 21 days of type 1 diabetes diagnosis. When analyzing the levels of autoantibodies, IAA, GADA, and IA-2A, levels were similar between ethnicities, and ZnT8A levels were higher in HW than NHW children (Fig. 1C). The majority of children had multiple islet autoantibodies independent of ethnicity (Table 1), and the profiles in terms of combinations are very similar between NHW and HW children (Table S1 (13)).

### Frequency of Type 1 Diabetes Predisposing HLA Alleles

As there were not robust differences in islet autoantibodies between our cohorts, we then evaluated HLA class II alleles in a subset of patients (n = 537 total) as specific DR and DQ alleles confer strong genetic risk for type 1 diabetes development (23, 24). The NHW group (n = 436) had a mean age at diagnosis of 12.3 ± 3.7 years, A1c = 12.3 ± 2.5%, 38.5% female, 55.8% DKA, and 58.9% had ≥3 positive autoantibodies, while the HW group (n = 101) had a mean age at diagnosis of 11.8 ± 3.8 years, A1c = 12.3 ± 2.1%, 46.5% female, 66% DKA, 66.3% had ≥3 positive autoantibodies. We observed a remarkably high prevalence of HLA-DR4 and DQ8 alleles, which are in linkage disequilibrium, among HW children, 82% and 79% respectively (Table 2). This was higher than the prevalence of these alleles in NHW children (70% for DR4, *P* = .014 comparing NHW with HW and 61% for DQ8, *P* < .001), and much higher than in a general Hispanic population (Table 3) (22). As a result of the high DR4-DQ8 prevalence, DR3 and DQ2 were lower in HW children (Table 2). DR4, DQ8, DR3, and DQ2 alleles had similar distributions with their corresponding haplotypes and genotypes (Table 2). Next, we aimed to validate the high frequency of DR4 and DQ8 alleles in a separate set of HW children. DNA samples from a randomly selected 100 HW children (mean age at diagnosis of 9.9 ± 3.7 years, A1c = 12.1 ± 2.3%, 46% female, 63.2% DKA, 66.0% had ≥3 positive autoantibodies) were HLA typed in a validation cohort, which replicated our findings for DR4 and DQ8, 81% and 80% respectively, in the initial HW cohort (Table 2).

**Table 2.** Type 1 diabetes risk HLA class II types by ethnicity

HLA type	Non-Hispanic White (N = 436)	Hispanic White initial cohort (N = 101)	*P* value	Hispanic White validation cohort (N = 100)
**DR and DQ alleles*^a^*, % (n)**				
DR4	70.0% (305)	82.1% (83)	.014	81.0% (81)
DQ8	61.0% (266)	79.2% (80)	<.001	80.0% (80)
DR3	48.4% (211)	31.7% (32)	.003	49.0% (49)
DQ2	48.2% (210)	30.7% (31)	.002	49.0% (49)
**DR-DQ haplotype, % (n)**				
DR4-DQ8	60.1% (262)	78.2% (79)	<.001	80.0% (80)
DR4-DQ7	12.4% (54)	2.0% (2)	.001	1.0% (1)
DR3-DQ2	48.2% (210)	30.7% (31)	.002	49.0% (49)
**DR-DQ genotype, % (n)**				
DR4-DQ8/X*^b^*	35.8% (156)	59.4% (60)	<.001	44.0% (44)
DR3-DQ2/X*^c^*	23.9% (104)	11.9% (12)	.007	13.0% (13)
DR4-DQ8/DR3-DQ2	24.3% (106)	18.8% (19)	.296	36.0% (36)
DRX/X*^d^*	16.1% (70)	9.9% (10)	.124	7.0% (7)

^*a*^DQ8 consists of DQA1*03:01-DQB1*03:02 and DQA1*03:03-DQB1*03:02, DQ2 is DQ2.5 and consists of DQA1*05:01-DQB1*02:01, and DQ7 is DQ7.3 and consists of the haplotypes DQA1*03:01/03:03-DQB1*03:01/03:04.

^*b*^X excludes the DR3-DQ2 haplotype.

^*c*^X excludes the DR4-DQ8 haplotype.

^*d*^X excludes the DR4-DQ8 and DR3-DQ2 genotype.

**Table 3.** ORs for HLA class II alleles and haplotypes by ethnicity

HLA type*^a^*	T1D Non-Hispanic White (%)	Caucasian reference frequency (%)	*P* value	OR (95% CI)	T1D Hispanic White (%)	Hispanic reference frequency (%)	*P* value	OR (95% CI)
DR4	70.0%	32.3%	<.001	4.9 (3.9-6.1)	81.6%	44.0%	<.001	5.6 (3.9-8.2)
DQ8	61.0%	20.0%	<.001	6.3 (5.0-7.8)	79.6%	37.3%	<.001	6.6 (4.6-9.5)
DR3	48.4%	24.8%	<.001	2.8 (2.3-3.5)	40.3%	14.2%	<.001	4.1 (3.0-5.5)
DQ2	48.2%	24.8%	<.001	2.8 (2.3-3.5)	39.8%	35.6%	.248	1.2 (0.9-1.6)
DQ6	2.5%	26.6%	<.001	0.1 (0.04-0.13)	0%	15.9%	<.001	NA
DQ7	17.2%	33.7%	<.001	0.4 (0.2-0.4)	6.0%	40.1%	<.001	0.1 (0.05-0.17)
DR4-DQ8	60.1%	20.0%	<.001	6.0 (4.8-7.5)	79.1%	36.8%	<.001	6.5 (4.6-9.3)
DR4-DQ7	12.4%	12.0%	.809	1.0 (0.8-1.4)	1.5%	3.8%	.110	0.4 (0.1-1.1)
DR3-DQ2	48.2%	24.8%	<.001	2.8 (2.3-3.5)	39.8%	14.1%	<.001	4.0 (3.0-5.5)
DR15-DQ6	2.5%	26.6%	<.001	0.1 (0.04-0.13)	0.0%	11.8%	<.001	NA

Abbreviations: HLA, human leukocyte antigen; OR, odds ratio; T1D, type 1 diabetes.

^*a*^DQ8 consists of DQB1*03:02, DQ2 consists of DQB1*02:01, DQ6 consists of DQB1*06:02, and DQ7 consists of DQB1*03:01 or *03:04*.

**Figure 2.**
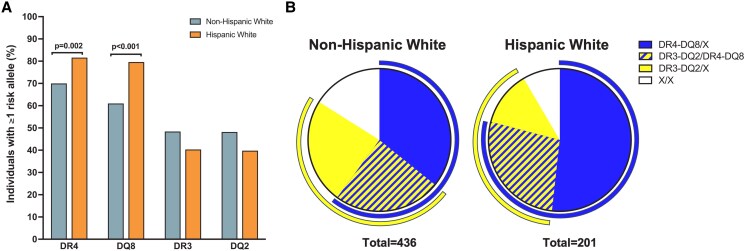
HLA class II DR and DQ types compared by ethnicity. (A) Prevalence of individuals having a type 1 diabetes risk HLA-DR4, DQ8, DR3, or DQ2 allele in non-Hispanic White children (blue bars) and Hispanic White children (orange bars); n = 201 for HW and n = 436 for NHW. (B) Prevalence of HLA genotypes having high-risk type 1 diabetes haplotypes by ethnicity. Curved blue line in the outer circle shows individuals with at least 1 DR4-DQ8 haplotype (NHW: 60.1% vs HW: 79.1%, *P* < .001 using a Fisher exact test). Curved yellow line in the outer circle shows individuals with at least 1 DR3-DQ2 haplotype (NHW: 48.2% vs HW: 39.8%, *P* = .049 using a Fisher exact test). DQ2 is defined as DQB1*05:01-DQB1*02:01.

Subsequently, we combined the HLA typing from the initial and validation HW cohorts to compare type 1 diabetes risk conferring HLA class II alleles to NHW children. As shown in Fig. 2A, there remained a remarkably high prevalence of DR4 and DQ8 in HW children (80%), while there was not a difference in DR3 and DQ2 alleles by ethnicity. Next, we compared the prevalence of these alleles with reference populations to assess risk for type 1 diabetes in both ethnicities. As expected, DR4, DQ8, DR3, and DQ2 had higher odds to be present in NHW and HW type 1 diabetes groups compared to Caucasian and Hispanic reference populations, respectively (Table 3). On the other hand, DQ6 (DQB1*06:02), which is known to confer dominant protection from type 1 diabetes development (25), was infrequent in both ethnic groups and had ORs ≤0.1 (Table 3). The prominent DR4-DQ8 haplotype had a high OR in both the HW group (OR 6.5, 95% CI 4.6-9.3) and the NHW group (OR 6.0, 95% CI 4.8-7.5). Although DR3-DQ2 was slightly more frequent in NHW children, both groups had high OR with HW (OR 4.0, 95% CI 3.0-5.5) and NHW group (OR 2.8, 95% CI 2.3-3.5). In addition to this individual level prevalence analysis, allele frequencies had a similar pattern, where DR4 and DQ8 were more prevalent in HW, while DR3 and DQ2 were slightly more prevalent in NHW children (Table S2 (13)).

As DR4 has multiple subtypes, we examined these by ethnicity. Although NHW children predominantly had DRB1*04:01 (45%) compared with only 17% in HW children, DRB1*04:01 had a high OR for both ethnicities (HW OR 5.2, 95% CI 3.3-8.0 and NHW OR 3.4, 95% CI 2.8-4.2) (Table S3 (13)). Interestingly, most of the other DR4 subtypes, including DRB1*04:02, *04:03*, *04:04*, *04:05*, *04:06*, and *04:07*, were more common in HW children than in NHW children and both ethnicities had comparable ORs for the presence of type 1 diabetes (Table S3 (13)).

When looking at the HLA-DR-DQ genotypes by ethnicity, there was again a predominance of DR4-DQ8 with and without DR3-DQ2 in HW compared with NHW children (Fig. 2B). There were similar percentages of the very high-risk genotype, DR4-DQ8/DR3-DQ2, between NHW and HW children (24% vs 27%, respectively). NHW children did have more DR3-DQ2 without DR4-DQ8 genotypes than HW children (24% vs 12%, *P* = .001 in Fig. 2B). Interestingly, children without either of the high-risk type 1 diabetes haplotypes, DR4-DQ8 or DR3-DQ2, were more prevalent in NHW than in HW (16.1% vs 8.1%, *P* = .009 in Fig. 2B), which indicates more NHW children have lower risk HLA genotypes than HW. When comparing islet autoantibody prevalence and numbers by HLA-DR-DQ genotypes, there are not distinct differences between ethnicities (Table S4 (13)).

Finally, to gain further insights into HLA risk for type 1 diabetes, we analyzed class I alleles in a subset of children. Several HLA class I alleles have been shown to confer risk in type 1 diabetes including A risk alleles (A*02, A*03, or A*24) and B alleles (B*15, *18*, *39*, *41*, *49*, *50*) (26, 27), and the frequencies of these alleles were not overly different between HW and NHW children (Table S5 (13)). Moreover, the groups were compared with reference Caucasian and Hispanic populations and showed similar ORs (Table S5 (13)). Notably, all the type 1 diabetes risk HLA class I alleles have lower ORs than those for class II (eg, DR4, DQ8, DR3, and DQ2). These data indicate that HLA class I diabetes-risk alleles are present in both NHW and HW children developing type 1 diabetes.

Taken together, our HLA class II and I typing data demonstrates high genetic risk within HW children developing type 1 diabetes, primarily conferred through HLA-DR4 and DQ8 alleles.

## Discussion

In this study, we focused on the clinical, immunologic, and genetic characteristics of HW children compared with NHW children who developed new-onset type 1 diabetes. There was a high prevalence of the HLA-DR4-DQ8 haplotype in HW children, present in 80%, a finding that we validated. Since the DR4-DQ8 haplotype confers the highest genetic risk for type 1 diabetes, its predominance in HW children may suggest genetically influenced disease pathogenesis. This genetic predisposition combined with increased exposure to environmental factors may contribute to the rising annual incidence rates of type 1 diabetes in the Hispanic population (3-5). Moreover, NHW children had haplotypes other than DR4-DQ8 or DR3-DQ2 2-fold more than HW children indicating a more homogeneous genetic risk profile among the latter group. The SEARCH for Diabetes in Youth study partially typed HLA class II alleles for *DRB1* and *DQB1*, showing that the DRB1*04:01 allele was more prevalent in NHW than in Hispanic children, while DRB1*04:04 and DRB1*04:07 were predominant in Hispanic children (10). Our findings from high-resolution HLA typing of all the polymorphic HLA class II genes supports these results and reveals that all major DR4 subtypes outside of DRB1*04:01 are more common in HW children along with the DQ8 allele. Additionally, smaller studies have found a higher prevalence of DQ8 in Hispanic vs non-Hispanic children with type 1 diabetes (28, 29). Accumulating evidence indicates that Hispanic and HW children with type 1 diabetes are more likely to have the DR4-DQ8 haplotype than NHW youth and the general Hispanic population.

Incorporating genetic differences from ethnic groups may facilitate the development of targeted interventions aimed at modulating immune responses and preserving pancreatic beta cell function in genetically predisposed high-risk populations. In this regard, HLA class II alleles may influence individual responses to immunomodulatory therapies, as evidenced by studies showing that the presence of DR4 were associated with better treatment responses when the anti-CD3 monoclonal antibody, teplizumab, was used to delay the clinical onset of type 1 diabetes (30). Additionally, there are several immunotherapies under investigation targeting specific HLA class II molecules including proinsulin and IA-2 peptide immunotherapy for HLA-DR4 (31, 32), methyldopa blocking HLA-DQ8 (33), and the GAD-alum vaccine with beneficial effects on individuals having the HLA-DR3-DQ2 haplotype (34, 35). Thus, tailoring treatment approaches based on an individual's HLA class II genotype may enhance therapeutic efficacy which necessitates ethnic-specific considerations to account for the genetic diversity observed in type 1 diabetes populations.

To the best of our knowledge, our study is the first to measure all 4 biochemical islet autoantibodies in HW children at type 1 diabetes onset. This comprehensive autoantibody profiling in children showed that Hispanic ethnicity does not appear to significantly influence autoantibody profiles at diagnosis. Redondo and colleagues showed no difference for IAA and GADA between Hispanic and NHW children at diabetes onset (6). The Pediatric Diabetes Consortium measured GADA and IAA, finding similar results in Hispanic children (82% and 59%, respectively) to our results reported here for HW children (73% for GADA and 51% for IAA) (11). Similarly, islet autoantibody prevalence in the SEARCH study was ∼70% for GADA and ∼80% for IA-2A without a difference between NHW and Hispanic children (10). Our results contribute to a comprehensive understanding of islet autoantibody profiles, revealing comparability between numbers and prevalence of each autoantibody between NHW and HW children.

Another significant finding from our study was the higher prevalence of DKA across all age groups in HW children. A number of previous studies have shown a higher prevalence of DKA in ethnic minorities (6-9); however, we show for the first time that this discordance spanned nearly all ages across childhood and adolescence. Screening for type 1 diabetes risk can decrease DKA incidence, result in better long-term glycemic control, and increase quality-adjusted life-years (12). However, there are barriers to be addressed in screening Hispanic populations. Hispanics were less likely to screen for type 1 diabetes prevention trials (36), and ethnic minorities were less likely to return for confirmation and monitoring visits following screening compared to NHW individuals (37). Proactive measures such as developing culturally appropriate interventions and support services that address the specific needs and challenges faced by these groups and their families may overcome these barriers to screening for type 1 diabetes risk.

Our study is not without limitations. First, we collected self-reported race and ethnicity, which is common in clinical practice; however, we did not assess genetic ancestry. To overcome this limitation and increase the concordance between genetic and self-reported race/ethnicity, we limited our study group to White individuals. Future studies will be done to evaluate genetic ancestry and genes outside of HLA class I and II alleles, which will facilitate the development of type 1 diabetes genetic risk scores for Hispanic ethnicities (38). Second, we did not collect data on socioeconomic determinants of health, which are known to be associated with DKA at diabetes onset (9). Third, we were unable to study other races and ethnicities with new-onset type 1 diabetes due to an overall lower incidence and numbers. Recently, genetic risk has been evaluated in type 1 diabetes with an African ancestry that enabled the development of an African-specific genetic risk scores (39).

In conclusion, we showed that HW children have comparable islet autoantibody profiles to NHW youth at type 1 diabetes onset but have a high prevalence of the HLA-DR4-DQ8 haplotype, as frequent as 4 out of 5 HW children. The HW population has genetic differences compared to NHW that can be used to identify children that would benefit from immune monitoring to detect early islet autoimmunity and potentially delay type 1 diabetes onset.

## Data Availability

Original data generated and analyzed during this study are included in this published article or in the data repositories listed in References.

## Abbreviations

BMI: body mass index
DKA: diabetic ketoacidosis
GADA: glutamic decarboxylase autoantibody
HLA: human leukocyte antigen
HW: Hispanic White
IAA: insulin autoantibody
IA-2A: tyrosine phosphatase-related islet antigen-2 autoantibody
NHW: non-Hispanic White
ZnT8A: zinc transporter 8 autoantibody
